# Recovery Patterns After Epineuroclasis and Endoneuroclasis Stretch Injuries in the Rat Median Nerve

**DOI:** 10.3390/biomedicines14071626

**Published:** 2026-07-20

**Authors:** Christoph A. Schroen, Damien Laudier, Philip Nasser, Paul J. Cagle, Michael R. Hausman

**Affiliations:** 1 Leni & Peter W. May Department of Orthopaedic Surgery, Icahn School of Medicine at Mount Sinai, New York, NY 10029, USA; damien.laudier@mssm.edu (D.L.);; 2 Medical Faculty Heidelberg, Heidelberg University, 69120 Heidelberg, Germany

**Keywords:** nerve injury, animal model, histology, endoneurium, epineurium, neuroma

## Abstract

**Background/Objectives:** This study used the neuroclasis nerve injury model to compare the long-term histomorphological, cellular, and inflammatory consequences of epineuroclasis and endoneuroclasis stretch injuries. **Methods:** Upon IACUC approval, 45 male Sprague-Dawley rats were allocated to 6 injury groups: an epineuroclasis and an endoneuroclasis group with follow-up at 2 weeks (n = 6 each), 6 weeks (n = 8 each), and 12 weeks (n = 8 and 9). Rats underwent left median nerve stretch to epineuroclasis or endoneuroclasis thresholds using load–deformation curve monitoring. Nerves were harvested at follow-up for qualitative histology and immunohistochemistry (H&E, NF200, S100, CD68, and Glut-1). **Results:** At 2 weeks, both injury levels demonstrated minimal NF200 staining, consistent with Wallerian degeneration. Epineuroclasis resulted in persistent epineurial rupture with exposed endoneurial tubes at 2, 6, and 12 weeks, indicating no structural restoration of the epineurium. At 12 weeks, axonal regrowth was observed primarily in regions with an intact epineurium and was limited in regions lacking epineurial coverage. Endoneuroclasis was associated with neuroma formation in 16/17 nerves (13/14 in-continuity) at 6 and 12 weeks, characterized by hypervascularity, aberrant fascicle formation, diffuse axonal sprouting, Schwann cell hypercellularity, and sustained macrophage infiltration. **Conclusions**: Epineurial integrity appears more important for axonal regeneration than previously thought. Persistent epineurial disruption was associated with limited axonal regrowth, whereas endoneurial disorganization was associated with neuroma formation despite macroscopic nerve continuity at time zero. Understanding the regenerative consequences of distinct degrees of connective tissue damage after stretch injury may help guide the development of tools that could diagnose these injuries early and potentially predict recovery after nerve injury.

## 1. Introduction

Peripheral nerve injuries are often devastating injuries that result in considerable long-term disability and pain for patients [1,2]. Persistent neurological deficits, loss of employment and an inability to do many activities of daily living place a significant burden on these often young and otherwise healthy patients. Many stretch injuries are in-continuity injuries [3], which are particularly challenging to treat as the extent of damage often cannot be determined in the acute setting [4]. Without knowing whether a nerve will heal on its own, physicians cannot determine whether a nerve injury would benefit from surgical interventions in the acute setting, while delaying nerve surgery often results in considerably worse outcomes [5,6]. For decades, physicians and scientists put great efforts into solving this clinical dilemma [7]. So far, these efforts have not led to the development of diagnostic approaches that could predict recovery (or lack thereof) after nerve injury.

In order to determine the recovery potential of an in-continuity nerve injury, it is paramount to understand how damage to the specific anatomical structures of a nerve impacts its ability to regenerate. The development of novel imaging modalities requires a particularly thorough understanding of the long-term consequences of distinct structural damage configurations in order to derive prognostic information from the structural damage visualized via nerve imaging. Previous efforts to develop such diagnostic tools may have been limited by a poor understanding of the sequence of mechanical and structural failure along which nerves fail during stretch injury [8]. A novel in vivo animal model was recently developed with the goal of inducing specific degrees of nerve injury by identifying specific mechanical failure events along the nerve’s load–deformation curve during stretching in real time [8]. This work resulted in a novel classification of nerve stretch injuries, the neuroclasis classification, which includes two specific degrees of mechanical and structural failure called epineuroclasis and endoneuroclasis [8]. Epineuroclasis, the first event of mechanical failure during stretching, was marked by disruption of the nerve’s epineurium, exposing the endoneurial core while endoneurial tubes remained intact. Endoneuroclasis, the second failure event, was marked by extensive endoneurial tube thinning and disorganization as well as disruption of endoneurial vasculature although nerves remained macroscopically in-continuity. The neuroclasis model and classification system enable investigations into the ability of diagnostic tools to grade injury severity and discern specific structural damage configurations. A recent study demonstrated the ability to identify and distinguish acute epineuroclasis and endoneuroclasis injuries intraoperatively using Second Harmonic Generation nerve imaging, which visualizes collagen fibers at a microscopic scale without damaging the nerve [9]. These findings support the feasibility of structural nerve injury grading in the acute setting. However, the clinical value of such grading depends on whether these acutely defined structural injury patterns correspond to distinct regenerative trajectories and long-term recoverability.

The epineurium and endoneurial tubes play different roles in nerve regeneration. While disruption of the outer connective tissue envelope may still permit axonal regeneration if the endoneurial architecture remains preserved, disruption of the endoneurial compartment may impair directed axonal regrowth and thus result in disorganized axonal growth and ultimately neuroma formation. Accordingly, subsequent rodent studies demonstrated recovery of motor function by twelve weeks after epineuroclasis injury, whereas endoneuroclasis injury resulted in persistent functional loss and traumatic neuroma formation [10,11]. However, functional outcomes alone are not sufficient to define the cellular and tissue-level differences between a likely-to-recover and an unlikely-to-recover in-continuity stretch injury. Therefore, it remains unknown how epineuroclasis and endoneuroclasis injuries differ in their longitudinal patterns of structural regeneration. This study aimed to characterize the short-, mid-, and long-term histologic changes following neuroclasis injuries in order to determine how distinct structural damage configurations influence the regenerative response following in-continuity nerve injuries.

## 2. Methods

### 2.1. Study Overview

This was a randomized controlled in vivo rat study comparing two mechanically defined median nerve stretch-injury grades, epineuroclasis and endoneuroclasis, with terminal histologic and immunohistochemical assessment at 2, 6, and 12 weeks. Upon IACUC approval, 47 twelve-month-old male Sprague-Dawley rats were allocated to 6 injury groups via simple randomization: an epineuroclasis (EP) and an endoneuroclasis (EN) injury group for follow-up at two weeks (n = 6 rats each), six weeks (n = 8 each) and twelve weeks (n = 8 and 9 rats). Two additional rats were used to harvest uninjured control tissue. Left median nerves were injured, right served as sham control. Male rats at twelve months were used due to their large size, facilitating careful microsurgical handling of nerves. Median nerves were harvested for histology at the respective follow-up time points and rats were euthanized.

### 2.2. Housing and Husbandry

Surgeries were performed in an accredited animal facility. Animals were housed individually in cages in a light- and temperature-controlled environment, with free access to food and water. All rats received subcutaneous buprenorphine (0.05–0.1 mg/kg) immediately before the surgery and twice daily for 72 h, including the preoperative dose. Rats were monitored for pain and distress each day post-injury until one week post-surgery. No adverse events were observed.

### 2.3. Nerve Surgery

Rats were anesthetized with 2.0% Isoflurane (Baxter Pharmaceutical Products, Deerfield, IL, USA) in 1 liter O_2_ per minute and prepared for surgery under sterile conditions. Following an incision of the skin and subcutaneous fascia, the flexor carpi radialis and flexor digitorum superficialis muscles were separated using microdissection tools. Median nerves of both forelimbs were surgically exposed over a length of approximately 2 cm and gently separated from surrounding tissue.

### 2.4. Nerve Injury Procedure

Left median nerves were stretched to one of two distinct levels of injury using the neuroclasis injury model [8]. Injury was defined as a rapid force reduction in the nerve’s load–deformation curve. A metal hook was attached to a load cell (Transducer Techniques, Temecula, CA, USA) and fixed to a servo-hydraulic material testing system (model 8872, Instron, Norwood, MA, USA). Load-cell data were used to generate load–deformation curves during the experiment in real time. Two blunt metal pins were fixed to a movable stage at a distance of 1 cm and were lowered onto the nerve, exposing 8.4 mm of nerve while securing it proximally and distally. The hook was gently positioned beneath the nerve, and elevated at a speed of 0.2 mm/second to stretch the nerve. Force–time curves were monitored in real time and the stretch was manually stopped immediately when an epineuroclasis or endoneuroclasis injury was observed, respectively. Epineuroclasis injury was achieved with a first drop of resistance force in the load–deformation curve, while endoneuroclasis injury was marked by a second steep decrease in force. Nerves were evaluated for macroscopic continuity after the stretch procedure.

### 2.5. Histology

Median nerves were harvested and fixed in 10% zinc-formalin for 48 h. Samples were processed using a hydrophobic acrylic resin [12]. Sections having a thickness of 5 μm were cut longitudinally from embedded blocks. De-plasticized sections were stained with Hematoxylin & Eosin (H&E) to visualize general tissue morphology. Immunohistochemical assays (Abcam Limited, Cambridge, UK) included chromogenic staining for Neurofilament 200 kDa (NF200 in red) with a toluidine-blue counterstain for nerve fibers, S100 (in brown) with a toluidine-blue counterstain for Schwann cells, CD68 (brown) with a toluidine-blue counterstain for macrophages indicating inflammation, and Glucose Transporter 1 (Glut-1 in purple) with a methyl-green counterstain to identify perineurial cells and intraneural metabolic activity [13,14,15]. Images were obtained using a Hamamatsu NanoZoomer S210 slide scanner (Hamamatsu Photonics, Hamamatsu City, Japan) and qualitatively analyzed.

Histological and immunohistochemical findings were assessed qualitatively. Longitudinal whole-nerve sections of every nerve and animal were reviewed across the injured segment with attention to regional differences between areas with preserved epineurial coverage, areas of epineurial disruption with exposed endoneurium, and neuroma tissue where present. H&E-stained sections were assessed for epineurial continuity, endoneurial tube organization, aberrant fascicle formation, intraneural hemorrhage, hypervascularity, fibrosis/scarring, fatty or muscle ingrowth, and neuroma-in-continuity formation. NF200-stained sections were assessed for the presence, regional distribution, and orientation of axons. S100-stained sections were assessed for Schwann-cell distribution and hypercellularity. CD68-stained sections were assessed for macrophage infiltration and digestion chamber formation. Glut-1-stained sections were assessed for peri-/epineurial staining patterns and regional intraneural metabolic activity.

## 3. Results

During the injury procedure, three of nine endoneuroclasis nerves in the twelve-week group suffered complete transection at the endoneuroclasis point, while six of nine endoneuroclasis nerves remained in-continuity.

### 3.1. Uninjured Nerve Histology

**Figure 1:**
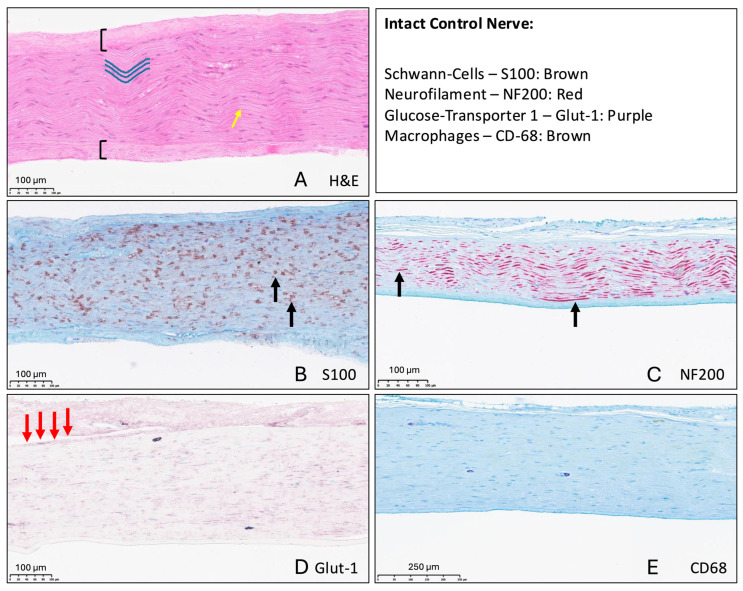
**Histologic and immunohistochemical appearance of intact rat median nerve.** Representative longitudinal sections of an uninjured rat median nerve demonstrating normal nerve architecture. H&E staining shows preserved epineurial (black brackets) and endoneurial organization with undulating endoneurial tubes (blue lines) and axons (yellow arrow) (**A**). S100 staining identifies Schwann cells (black arrows) within the endoneurium (**B**). NF200 staining demonstrates longitudinally oriented axons (black arrows) (**C**). Glut-1 staining demonstrates faint endoneurial staining and stronger peri-/epineurial staining (red arrows) (**D**). CD68 staining shows minimal macrophage presence in intact nerve tissue (**E**). Scale bars as shown.

Uninjured nerve samples demonstrated normal nerve architecture with the epineurium surrounding the endoneurial core. Endoneurial tubes and axons followed a characteristic undulating pattern, which serves as a physiological internal reserve length for stretching (Figure 1A) [16,17,18]. S100 staining visualized individual Schwann cells within the endoneurium (Figure 1B). NF200 staining marked nerve fibers longitudinally (Figure 1C), Glut-1 staining demonstrated subtle positive staining in the endoneurium and stronger staining of the peri- and epineurium (Figure 1D), and CD68 demonstrated only minimal macrophage infiltration in uninjured rat median nerves (Figure 1E).

### 3.2. Epi- and Endoneuroclasis Two Weeks After Injury

As both injury levels include disruption of the epineurium, nerves from both groups exhibited three zones along the nerve on histology at two weeks: a zone with an intact epineurium, an area showing epineurial disruption, and a region with an exposed endoneurial core.

**Figure 2:**
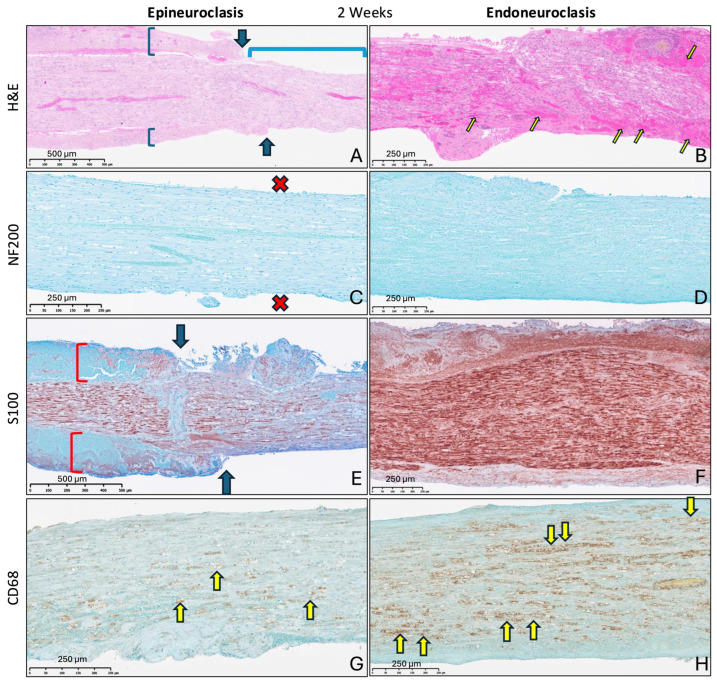
**Histologic and cellular findings two weeks after epineuroclasis and endoneuroclasis injury.** Representative longitudinal sections of epineuroclasis and endoneuroclasis nerves two weeks after stretch injury. H&E staining demonstrates persistent epineurial disruption (dark blue brackets show epineurium, arrows show epineurial disruption) and exposed endoneurial tissue (light blue bracket) after epineuroclasis (**A**), while endoneuroclasis shows greater endoneurial disorganization and intraneural hematoma formation (yellow arrows) (**B**). Red crosses indicate lack of an epineurium in Figure 2C, and NF200 staining demonstrates near-complete loss of axonal staining in both injury groups, consistent with Wallerian degeneration (**C**,**D**). S100 staining demonstrates Schwann-cell presence after epineuroclasis, with reduced staining in exposed endoneurial regions (epineurium marked by red brackets, disruption marked by blue arrows) (**E**), and more extensive Schwann-cell infiltration after endoneuroclasis (**F**). CD68 staining demonstrates macrophage infiltration (yellow arrows) after both injury grades, with more prominent inflammatory changes after endoneuroclasis (**G**,**H**). Scale bars as shown.

Epineuroclasis at two weeks:

All signs of an acute epineuroclasis injury were also present at two weeks, including epineurial disruption (Figure 2A) and straightened endoneurial tubes indicating plastic deformation. Periaxonal hemorrhage was observed in the exposed endoneurium. NF200 staining revealed a near-complete lack of axons, which is consistent with Wallerian degeneration at two weeks (Figure 2C). Marked invasion of the endoneurium by Schwann cells was visible in areas where the epineurium remained continuous (Figure 2E). However, S100 positivity was considerably reduced distal to the epineurial disruption, with only subtle staining in the exposed endoneurial core (Figure 2E). CD-68 staining depicted inflammation in the form of macrophage infiltration in all zones of epineuroclasis nerves at two weeks (Figure 2G).

Endoneuroclasis at two weeks:

At two weeks, endoneuroclasis nerves exhibited a disrupted epineurium and an exposed endoneurial core with highly disorganized endoneurial tubes and ubiquitous intraneural hematoma formation, similar to acute endoneuroclasis injuries (Figure 2B). Newly formed vasculature was visible within the epineurium two weeks after endoneuroclasis. An almost complete lack of NF200 staining at two weeks was consistent with Wallerian degeneration (Figure 2D). Extensive infiltration of the endoneurial compartment by Schwann cells was observed in S100-stained sections (Figure 2F). Large numbers of macrophages and digestion chambers were present throughout CD-68 stained endoneuroclasis nerve sections (Figure 2H).

In summary, the structural damage that is induced by acute epi- and endoneuroclasis injury appeared largely unchanged at two weeks. Both injuries resulted in Wallerian degeneration. Endoneuroclasis injury was followed by formation of new epineurial vasculature at two weeks. Schwann-cell migration was limited where the epineurium was disrupted following epineuroclasis. Early morphological changes and cellular responses to injury were overall more drastic after endoneuroclasis compared to epineuroclasis injury at two weeks.

### 3.3. Epineuroclasis at Six and Twelve Weeks

**Figure 3:**
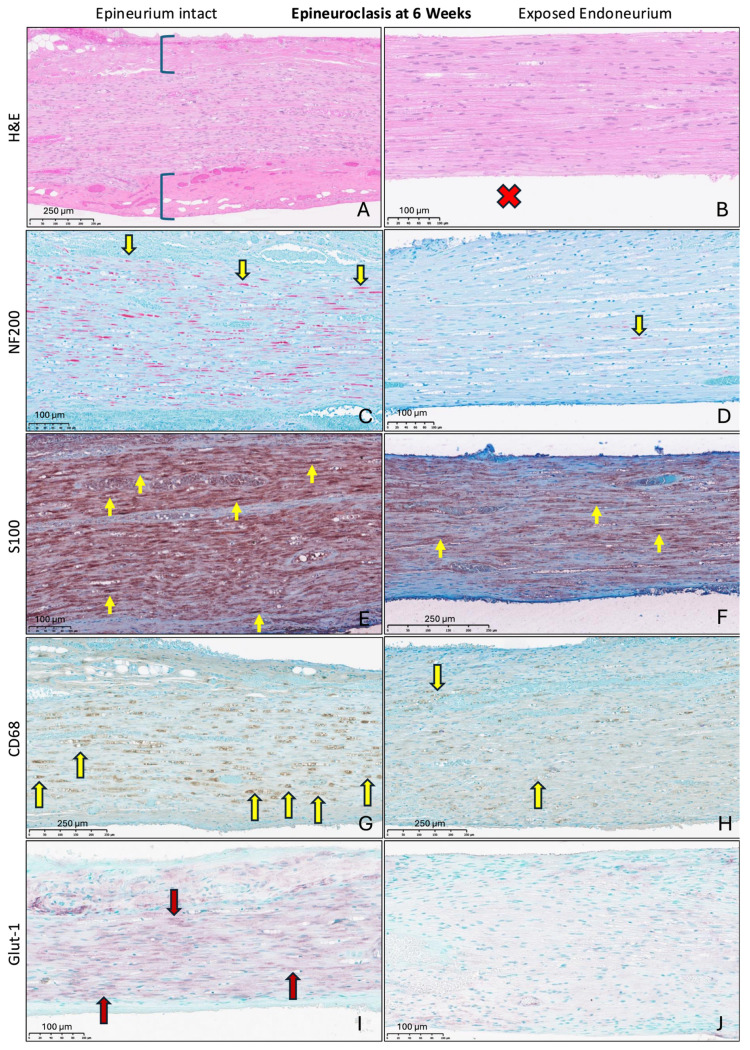
**Regional differences in structural regeneration six weeks after epineuroclasis.** Representative longitudinal sections of epineuroclasis nerves six weeks after injury comparing regions with preserved epineurial coverage (blue brackets Figure 3A) and regions with exposed endoneurium (Figure 3B, red x indicates lack of epineurium). H&E staining demonstrates persistent epineurial disruption and straightened exposed endoneurial tubes (**A**,**B**). NF200 staining shows greater axonal regeneration in regions with intact epineurium, whereas exposed endoneurial regions show limited neurofilament staining (yellow arrows) (**C**,**D**). S100 staining (yellow arrows) is more prominent in regions with preserved epineurial coverage than in exposed endoneurial regions (**E**,**F**). CD68 staining demonstrates macrophage infiltration (yellow arrows), particularly in regions with intact epineurial coverage (**G**,**H**). Glut-1 staining is more prominent in covered endoneurial regions (red arrows) and reduced in exposed endoneurial tissue (**I**,**J**). Scale bars as shown.

The epineurium remained disrupted six weeks after epineuroclasis and contained newly formed blood vessels proximal to its disruption (Figure 3A). Endoneurial tubes and axons remained straight within the exposed endoneurial core and exhibited no major morphological changes at six weeks compared to two weeks (Figure 3B). NF200 staining showed regeneration of nerve fibers where the epineurium was intact (Figure 3C), while only minimal staining for neurofilaments was present in the exposed core (Figure 3D). Similarly, S100 staining was more prominent proximal to the epineurial disruption (Figure 3E,F). Macrophage infiltration was considerably greater in areas with an intact epineurial sheath (Figure 3G,H) and indicated ongoing inflammatory processes six weeks after epineuroclasis. Metabolic activity in the endoneurium, marked by Glut-1 positivity, was also observed primarily where the epineurial sheath was intact (Figure 3I), while the exposed endoneurium demonstrated only subtle Glut-1 positivity (Figure 3J).

**Figure 4:**
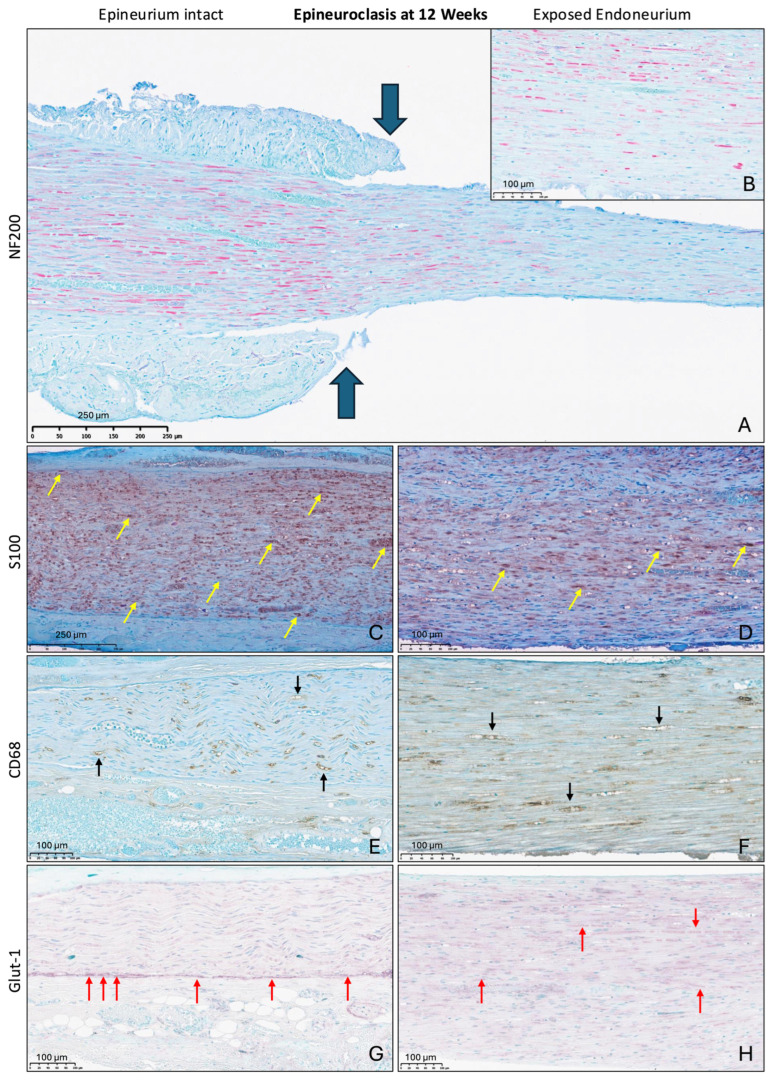
**Regional differences in structural regeneration twelve weeks after epineuroclasis.** Representative longitudinal sections of epineuroclasis nerves twelve weeks after injury. NF200 staining demonstrates axonal regeneration primarily in regions with preserved epineurial coverage (epineurial disruption marked by arrows) (**A**), while exposed endoneurial regions show comparatively limited axonal staining (**B**). S100 staining appears modest in both covered and exposed regions at twelve weeks (yellow arrows) (**C**,**D**). CD68 staining demonstrates residual macrophage presence (black arrows) despite overall reduction compared with earlier time points (**E**,**F**). Glut-1 staining (red arrows) demonstrates faint, more diffuse staining patterns at twelve weeks, consistent with decreased metabolic activity compared with six weeks (**G**,**H**). Endoneurial Glut-1 staining was more prominent where the endoneurium was exposed. Scale bars as shown.

The epineurium remained disrupted at twelve weeks. Endoneurial tubes and axons returned to follow an undulating course proximal to the epineurial disruption, indicating recovery from plastic deformation in this area. In contrast, no major morphological differences were observed in the exposed endoneurial core between two weeks, six weeks and twelve weeks, indicating a lack of structural recovery in this area. NF200 staining remained similarly limited in the exposed endoneurium compared to areas with an epineurial sheath at twelve weeks (Figure 4A,B). S100-staining appeared more modest at twelve weeks (Figure 4C,D). Similarly, CD68 positivity returned to mild levels at twelve weeks but remained higher than baseline levels with remaining macrophages visible in all areas of the nerve (Figure 4E,F). Glut-1 staining patterns normalized and exhibited faint ubiquitous staining throughout the endoneurium, demonstrating a potential decrease in metabolic activity compared to six weeks (Figure 4G,H).

### 3.4. Endoneuroclasis at 6- and 12-Weeks

**Figure 5:**
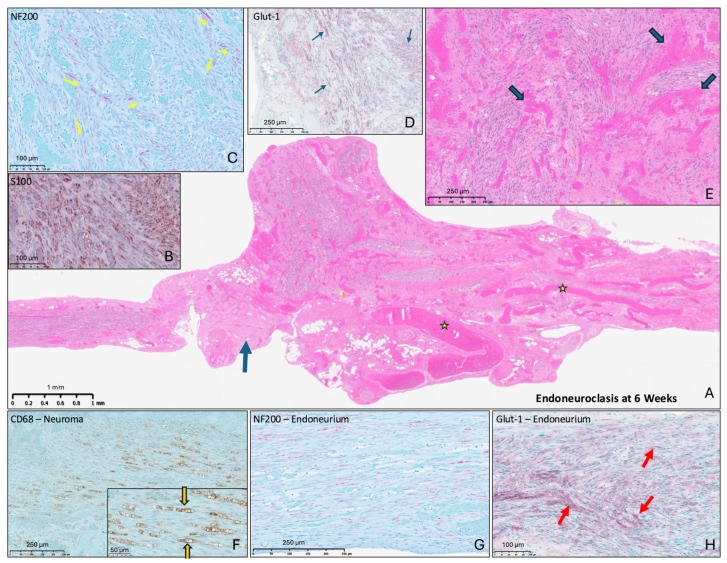
**Neuroma in-continuity formation six weeks after endoneuroclasis.** Representative longitudinal sections of endoneuroclasis nerves six weeks after injury. H&E staining demonstrates neuroma in-continuity formation with marked architectural distortion, muscle ingrowth (blue arrow) and hypervascularity (yellow stars in (**A**), blue arrows in (**E**)) within neuroma tissue (**A**,**E**). S100 staining shows prominent Schwann-cell hypercellularity within the neuroma (**B**). NF200 staining demonstrates disorganized axonal sprouting within neuroma tissue and adjacent endoneurial regions (yellow arrows indicate axonal orientation) (**C**,**G**). Glut-1 staining (blue arrows in (**D**), red arrows in (**H**)) demonstrates increased metabolic activity within neuroma and endoneurial tissue (**D**,**H**). CD68 staining demonstrates macrophage infiltration (orange arrows) within the neuroma, consistent with ongoing inflammation (**F**). Scale bars as shown.

**Figure 6:**
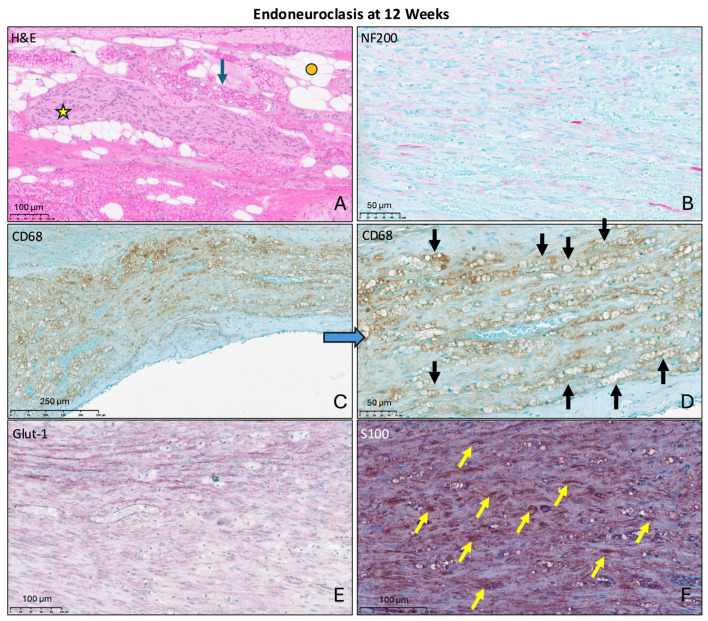
**Chronic neuroma-associated remodeling twelve weeks after endoneuroclasis.** Representative longitudinal sections of endoneuroclasis nerves twelve weeks after injury. H&E staining demonstrates persistent neuroma architecture with aberrant fascicle formation (yellow star), hypervascularity (small blue arrow), fatty infiltration (orange circle), and tissue disorganization (**A**). NF200 staining demonstrates disorganized axonal sprouting within neuroma tissue (**B**). CD68 staining demonstrates persistent macrophage infiltration (black arrows), consistent with chronic inflammation (**C**,**D**). Glut-1 staining remains prominent, indicating sustained metabolic activity within neuroma tissue (**E**). S100 staining demonstrates persistent Schwann-cell hypercellularity (yellow arrows) (**F**). Scale bars as shown.

Six weeks after endoneuroclasis, 8/8 nerves formed a neuroma-in-continuity (Figure 5A). Similarly, 5/6 intact endoneuroclasis nerves formed a neuroma-in-continuity at twelve weeks. Overall, 16/17 endoneuroclasis injuries resulted in a neuroma at six and twelve weeks, with 13/14 macroscopically continuous nerves forming a neuroma. Neuromas displayed extensive hypervascularity (Figure 5E), aberrant fascicle formation (Figure 6A), intraneural scarring, fatty infiltration and muscle ingrowth. NF200 staining revealed highly disorganized nerve fibers and diffuse axon overgrowth (Figure 5C,G and Figure 6B), accompanied by massive Schwann-cell infiltration shown via S100 staining (Figure 5B and Figure 6F). Presence of macrophages throughout neuroma tissue (Figure 5F) indicated a prolonged, chronic inflammatory response at 12 weeks (Figure 6C,D), potentially indicating ongoing degeneration and regeneration attempts [19,20]. Prominent Glut-1 positivity at 6 (Figure 5D) and 12 weeks (Figure 6E) indicates chronically increased metabolic activity and highlights potentially ongoing regeneration attempts [13,14].

### 3.5. Summary of Histology Findings

In summary, epineuroclasis and endoneuroclasis exhibited distinct morphological and cellular recovery paths. Epineuroclasis nerves exhibited persistent epineurial disruption at all timepoints, indicating an inability of the disrupted epineurial sheath to regenerate. Furthermore, the exposed endoneurial core showed only limited axonal regeneration as well as comparatively low Schwann-cell and macrophage infiltration following epineuroclasis.

Endoneuroclasis injury resulted in formation of traumatic neuromas-in-continuity, and led to gross hypervascularity, chronic Schwann cell and macrophage infiltration and axonal overgrowth. The findings described for each group and timepoint were consistent among animals in these respective groups.

## 4. Discussion

This study investigated the short-, mid-, and long-term histologic and morphologic changes following two distinct degrees of nerve stretch injury in rats to characterize how these damage configurations influence the structural regenerative response of in-continuity nerve injuries. Both injury grades, epineuroclasis and endoneuroclasis, experienced Wallerian degeneration at 2 weeks. Epineuroclasis injury caused persistent epineurial discontinuity and regionally limited Schwann-cell migration and axonal regeneration where the endoneurium was exposed. Endoneuroclasis injury resulted in a neuroma-in-continuity with chronic inflammation and disorganized endoneurial contents. These results indicate that neuroclasis injuries have distinct structural regeneration trajectories and outcomes. These findings further show that epineurial disruption may limit axonal regeneration considerably and that endoneurial disorganization likely results in an injury phenotype that is unlikely to recover naturally and thus has a poor prognosis. The neuroclasis injury model may be used to investigate the prognostic abilities of novel diagnostic tools by identifying these injury grades at time zero.

### 4.1. The Neuroclasis Injury Model

The neuroclasis framework describes mechanically and structurally defined degrees of nerve stretch injury. Less severe traumatic nerve damage is often classified as neurapraxia and may be limited to demyelination with a high likelihood of full recovery, whereas the prognosis of axonopathic injury, often classified as axonotmesis, depends on whether the extent of structural damage permits axonal regeneration [21,22]. As Wallerian degeneration occurred in both injury levels, both epineuroclasis and endoneuroclasis injuries lie on the axonotmesis injury spectrum, exceeding the degree of neurapraxia. The difference between neuroclasis injuries and prior classifications lies in the sequence of structural disintegration during stretching and their implications for recovery [23,24].

This study demonstrated that the neuroclasis model of rat median nerve stretch injury produces two injury grades that follow distinct recovery trajectories and result in different long-term outcomes. Prior research showed that rat forepaw grip strength fully returns to baseline strength 12 weeks after epineuroclasis injury, while grip strength after endoneuroclasis injuries remained fully lost at 12 weeks [10,11]. Taken together, these findings render the neuroclasis model a promising preclinical platform for inducing mechanically and structurally distinct in-continuity nerve injuries. Future research will be needed to confirm whether the same damage sequence and injury grades occur during stretch injury of human nerves.

### 4.2. Implications of Epineuroclasis

Epineuroclasis injury resulted in Wallerian degeneration at 2 weeks but overall demonstrated successful axonal regeneration at 12 weeks. However, the epineurium failed to regenerate and remained disrupted at every follow-up timepoint. Schwann cell migration, axonal regeneration and recurrence of endoneurial tube undulations were reduced in areas where the endoneurial core was exposed. Van Neerven et al. showed that epineurial fibroblasts promote Schwann cell migration and provide a large reservoir of pro-regenerative cytokines [25], such as vascular endothelial growth factor (VEGF) or nerve growth factor (NGF). This potentially pro-regenerative effect of epineurial fibroblasts on injured nerves may help explain why epineurial disruption may have a deleterious effect on structural regeneration, as observed in this study. The integrity of the outer connective tissues may thus be more important for successful structural recovery than previously anticipated. Although preservation of endoneurial tubes remains central to directed axonal regeneration, the limited regeneration observed within exposed endoneurial regions suggests that epineurial integrity may also be required to maintain a permissive environment for organized nerve repair. Epineurial disruption may thus represent a therapeutically relevant aspect of in-continuity nerve injury. A recent study investigated whether the torn epineurium can be replaced using an amniochorionic membrane allograft nerve wrap following epineuroclasis injuries and found earlier functional recovery in rats treated with a nerve wrap [26]. Future studies are needed to determine whether restoring or protecting the outer connective tissue envelope improves regeneration in human patients.

### 4.3. Implications of Endoneuroclasis

Endoneuroclasis injury is marked by mechanical failure and disorganization of endoneurial tubes, as well as disruption of endoneurial vasculature while nerves remain macroscopically in-continuity in most cases [8]. Endoneuroclasis nerves underwent Wallerian degeneration at 2 weeks, and almost every endoneuroclasis injury resulted in a traumatic neuroma at 6 and 12 weeks with diffuse axonal sprouting, aberrant fascicle formation, hypervascularity, muscle and fatty ingrowth, Schwann-cell hypercellularity, and sustained macrophage infiltration. The endoneurial tubes are the structural framework along which Schwann cells align to form bands of büngner and along which damaged axons regenerate [27]. The observed pattern of extensive Schwann cell and macrophage proliferation at 6 and 12 weeks suggest ongoing but misdirected regeneration attempts resulting in chronic inflammation and misdirected neurite outgrowth [13,28,29]. This is important because macroscopic continuity can be misleading as the extent of structural damage remains unknown [4]. A nerve may remain “in continuity” but still be unlikely to recover, as the endoneurial compartment sustains damage that impairs guided axonal regrowth [30]. These rodent findings support the concept that endoneurial tube integrity is an important determinant of organized regeneration after stretch injury.

These histologic findings are particularly relevant for the development of structural imaging tools [31]. If acute imaging can distinguish epineuroclasis from endoneuroclasis, such imaging may offer prognostic information regarding the likelihood of organized regeneration in addition to morphologic classification. Future diagnostic tools such as Second Harmonic Generation (SHG) collagen imaging may be used to assess the extent of endoneurial damage in order to provide an accurate prognosis [32]. A recent study investigated whether epineuroclasis and endoneuroclasis injury could be visualized and distinguished intraoperatively using SHG imaging in vivo in rats [9]. Endoneuroclasis injuries exhibited considerably more endoneurial fiber breaks and disorganization, rendering SHG imaging a promising tool for identifying nerve damage that may be associated with poor outcomes, potentially warranting early surgical intervention.

### 4.4. Limitations

This study has several limitations. The neuroclasis model was performed in rat median nerves under controlled experimental conditions. Therefore, it remains unknown whether the same sequence of connective tissue failure, regenerative patterns, and prognostic implications occur in human in-continuity stretch injuries. Currently ongoing investigations aim to determine whether neuroclasis injuries similarly occur in human peripheral nerves. Nonetheless, we believe that the conceptual findings of the present study are broadly translatable to human peripheral nerves.

Prior research presented the functional outcomes of neuroclasis injuries, while this study focused on structural and cellular regeneration patterns. As both this study and the referenced functional experiments were conducted by the same research team, we believe that interpreting these findings together is justified. Nonetheless, additional functional outcome measures, including assessments of sensory function, may provide valuable information on the long-term implications of neuroclasis injuries and should thus be included in future research.

Endoneuroclasis represents a severe mechanically defined injury state, and a minority of nerves progressed to complete transection during injury induction. Nonetheless, neuroma-in-continuity formation was also observed in the majority of macroscopically continuous endoneuroclasis nerves, and we believe that this limitation of the neuroclasis model lies within the acceptable limits of varying injury severity seen with other animal models as well [33].

This study used qualitative histological and immunohistochemical assessment rather than quantitative morphometry. Quantitative approaches such as axon counts, myelin thickness, fiber density, g-ratio, or percentage area staining can be highly informative when applied to studies specifically designed around those endpoints, particularly in cross-sectional nerve specimens. However, the present study focused on longitudinal architectural patterns across multiple morphologically distinct areas after mechanically defined connective tissue failure, including epineurial discontinuity, exposed endoneurial regions, regional differences in axonal regeneration patterns, and neuroma-in-continuity formation. No validated scoring system currently exists for grading epineuroclasis- and endoneuroclasis-associated structural remodeling in longitudinal whole-nerve sections. Future studies should use prospective quantitative morphometric analysis protocols to validate the qualitative patterns identified in this study.

Histologic assessment was performed at predefined follow-up time points and therefore cannot fully capture the dynamic sequence of degeneration, inflammation, Schwann-cell migration, and axonal regrowth within the same nerve over time. In addition, although immunohistochemistry demonstrated consistent morphologic patterns, these findings cannot by themselves establish causality between epineurial disruption, endoneurial disorganization, and impaired regeneration. This study intended to identify structural regeneration patterns for two distinct degrees of nerve stretch injury in rats, which was achieved. Based on these findings, future preclinical and clinical studies are needed to confirm the observed findings in larger animal models and ultimately in human patients.

## 5. Conclusions

Two mechanically defined nerve stretch injuries in rats, epineuroclasis and endoneuroclasis, demonstrate distinct histologic recovery trajectories despite shared early Wallerian degeneration. Epineuroclasis was associated with persistent epineurial disruption and regionally limited regeneration in exposed endoneurial regions, suggesting that epineurial integrity may be important for successful and organized axonal regeneration. In contrast, endoneuroclasis resulted in chronic inflammation, disorganized axonal sprouting, and neuroma-in-continuity formation despite macroscopic nerve continuity. These findings suggest that prognosis after in-continuity stretch injury is determined by the extent to which the connective tissue architecture remains capable of supporting organized axonal regeneration. This supports the use of the neuroclasis model for evaluating diagnostic tools that aim to identify prognostically meaningful structural injury patterns in the acute setting.

## Data Availability

The original contributions presented in this study are included in the article. Further inquiries can be directed to the corresponding author.
